# Assessing the Impact of Microplastic Filaments Contaminated with PAHs on *Mytilus coruscus* Larvae through Surface Contact

**DOI:** 10.3390/toxics11070554

**Published:** 2023-06-24

**Authors:** Jiji Li, Ji Huang, Yingying Ye, Jiayin Lü, Shuai Mao, Jie Bai, Pengzhi Qi, Baoying Guo, Chengkai Qu, Hongchen Jiang

**Affiliations:** 1 State Key Laboratory of Biogeology and Environmental Geology, China University of Geosciences, Wuhan 430074, China; 2 National Engineering Research Center for Marine Aquaculture, Zhejiang Ocean University, Zhoushan 316022, China; 3 Shengsi Marine Science and Technology Institute, Zhoushan 202450, China

**Keywords:** contaminated microplastics, PAHs, mussel planktonic larvae, *Mytilus coruscus*

## Abstract

In recent years, microplastics have been of great concern in environmental and health research. In field surgeries and laboratory investigations, research interests were focused on the retention of microplastics inside of animals by ingestion and the series of negative effects after that. However, such large plastic debris and filaments are hardly eaten by small animals, like zooplankton, planktonic larvae, etc. In this study, the surface contact between plastic filaments contaminated with polycyclic aromatic hydrocarbons (PAHs) and mussel pediveliger larvae has been investigated to figure out the effects of “non-digestive tract route of exposure” on subject animals. In a 1600 mL artificial seawater medium, high mortalities of mussel larvae were recorded after being exposed to two PAHs-contaminated (benzo[α]pyrene (BaP) and phenanthrene (Phe)) filaments for 5 days, 68.63% for BaP and 56.45% for Phe on average. We suggest that the surface contact was the dominant pathway to transfer PAHs from contaminated filaments to larvae and that the risk of contaminated plastic ropes transferring hydrophobic organic compounds (HOCs) to larvae in mussel aquaculture should be taken seriously.

## 1. Introduction

Microplastics (MPs) are ubiquitous in the environment, and their potential ecological risks have gained a lot of attention in environmental and ecological research in recent decades. Ecotoxicity is a component of the ecological risks research of MPs. The toxic effects of MPs were usually revealed by direct exposure to environmental organisms [1,2]. MPs are known to accumulate in various ecosystems, including marine, freshwater, and terrestrial environments, where they can impact the health and survival of a wide range of organisms. The toxic effects of MPs have been widely reported, but the mechanisms by which they affect different organisms remain unclear. One potential pathway is through ingestion, which is the primary pathway to induce toxic effects on animals, such as fish in water and earthworms in soil, being mistaken for food and leading to physical harm, blockage of digestive tracts, and even death. In this way, the effects of MPs are size-dependent. The size of MPs is defined within the range of 0.1–5000 µm [3], which means that larger MPs cannot be ingested by small animals, such as zooplankton in aquatic environments. Additionally, since phytoplankton are usually smaller than MPs, the effects of ingesting larger MPs are not seen in these organisms. Thus, investigating the pathway of transferring toxicity from bigger MPs to smaller organisms is worth studying but rarely reported.

Besides the toxicity of MPs on their own, extra toxicity from contaminants the MPs have absorbed can leach into water as a pollution vector. Of the various contaminants that can accumulate on MPs, hydrophobic organic chemicals (HOCs) are among the most concerning due to their potential for long-term effects on the health of aquatic organisms. HOCs, including polychlorinated biphenyls (PCBs) [4], polycyclic aromatic hydrocarbons (PAHs) [5], organochlorine pesticides (OCPs) [6], phenols, etc., which have hydrophobic functional groups, then lead to high octanol-water partition ratios (*K_ow_*) and very low water solubility. This makes them more likely to adsorb onto MPs and increases their persistence in the environment. They are the most common “hitch-hikers” on MPs. In marine environment, the sink of MPs fate, HOCs can be enriched on MPs that the concentrations of adsorbed HOCs are several orders of magnitude higher than ambient concentrations [7,8]. Driven by currents and waves, MPs show strong migration capacity, and may transport HOCs to clean water from contaminated areas. The contaminated MPs bring high ecotoxicological risk to marine organisms. As a result, the potential for ecological risks posed by MPs may extend beyond their immediate surroundings, with far-reaching effects on marine ecosystems. The potential for long-distance transport of MPs and associated contaminants highlights the need for greater understanding of the fate and transport of MPs in aquatic environments.

The toxic effects of contaminated MPs on larger organisms, including swimming fish, crustacea, mollusc, etc., have been widely reported [9,10], because the exposure pathway is typically the “mouth-digestive tract” way for animals mentioned above. However, the overwhelming majority of animals consist of zooplankton in sea water, which cannot ingest big MPs with millimeter-level sizes. The non-typical exposure pathway of contaminated MPs to zooplankton, including planktonic larvae of bigger animals, has not been deeply investigated. These smaller organisms are critical components of aquatic ecosystems, serving as a food source for larger organisms and playing key roles in nutrient cycling and primary productivity. Therefore, understanding the potential impact of MPs on smaller organisms is essential for a comprehensive understanding of the ecological risks posed by MPs.

The presence of MPs in the water column can also have indirect effects on the behavior, physiology and overall fitness of smaller organisms. For example, exposure to MPs can induce changes in feeding behavior, swimming performance and energy allocation in zooplankton and larval fish, which could ultimately impact their survival, growth and reproductive success. Furthermore, MPs can interact with natural particles and organic matter in the water column, potentially altering the availability and quality of food resources for filter-feeding organisms, such as bivalve mollusks. Consequently, the potential impact of MPs on the trophic dynamics and energy flow within aquatic ecosystems is an area of ongoing research and concern.

In summary, the ecological risks posed by MPs are complex and multifaceted, with potential direct and indirect effects on a wide range of organisms and ecosystem processes. The potential for size-dependent impacts, as well as the role of contaminant transfer and bioaccumulation through the food chain, are important areas of ongoing research. Moreover, the interactions between MPs and other environmental stressors, as well as the influence of physical properties on the toxicity and behavior of MPs in aquatic environments, are critical factors to consider when evaluating the overall ecological risks associated with MPs.

In this study, we aim to investigate the potential impact of PAH-contaminated microplastic filaments on the larval mussel *Mytilus coruscus*, an important species of bivalve mollusk in the coastal waters of China. We conducted a exposure experiment of MPs contaminated by PAHs, benzo[α]pyrene (BaP) and phenanthrene (Phe), to determine the effects of contaminated MPs through in vitro contact towards larval mussels. Through our research, we hope to improve our understanding of the potential impact of MPs on smaller organisms, and to provide a foundation for the development of effective management strategies to mitigate the ecological risks posed by MPs in aquatic environments. We can take steps to protect the health and biodiversity of aquatic ecosystems, ensuring their continued health and resilience for future generations.

## 2. Method and Materials

### 2.1. Chemicals and Larval Mussels

The chemicals used in this study included two PAHs: BaP and Phe. These PAHs were chosen due to their widespread occurrence in the marine environment and their known toxic effects on aquatic organisms. PAHs BaP and Phe (sublimed grade, purity ≥ 99.5%) were purchased from Sigma Aldrich Co., Saint Louis, MO, USA. Dimethyl sulfoxide (DMSO, purity ≥ 99.0%) was purchased from Sinopharm Chemical Reagent Co., Ltd., Shanghai, China. Microplastic filaments used in the study were made of polyethylene (PE) both because it is a commonly used material in aquaculture operations and has been shown to accumulate organic contaminants such as PAHs. PE fibers (Φ 0.37 mm) were purchased from Jiadiakni Fishing Gear Co., Ltd., Zaoyang city, Hubei, China. Salts used to prepare artificial seawater (ASW) [11] for larvae medium were also purchased from Sinopharm Chemical Reagent Co., Ltd., Shanghai, China. 

**Figure 1:**
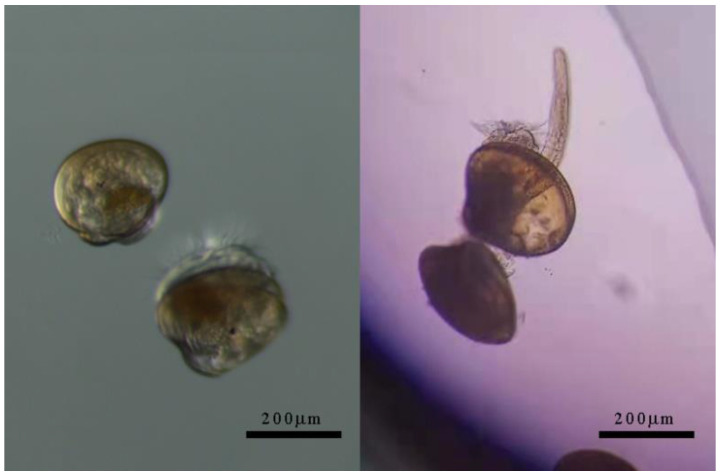
Observation of pediveliger larvae of mussel *Mytilus coruscus*.

Mussel reproducers were obtained from our breeding farm and sterilized by 30 mg/kg KMnO_4_ solution dipping bath for 5–10 min after the byssus had been clipped off. The adult male and female individuals were settled in a dry and cold (4 °C) environment for 6 h and transferred into seawater (18 °C, 30‰ salinity) at 130 cm depth. After 2–4 h, individuals which were at the beginning of ovulation and spermiation were removed and put into a clean beaker with seawater (18 °C, 26‰ salinity), respectively. The concentration of eggs was controlled at a level of 8–10 cells/mL and an excess of sperm cells was mixed into the eggs solution for fertilization. A few hours later, the superfluous sperm cells were filtered out from the fertilized eggs solution. Fertilized eggs were incubated in seawater (18 °C, 26‰ salinity; filtered and sterile) with a high oxygen concentration and grew to pediveliger larvae after 30–35 days. There were also three other stages during this period: trochophore, D-shape larvae and umbo larvae. Microalgae chrysophyceae was fed starting at the D-shape larvae stage. At the umbo larvae stage, a small quantity of diatom and platymonas were added into mussel’s diet. The seawater with aquaculture waste was replaced with clean seawater once every one or two days. The foot was observed at the pediveliger larvae stage, and the larvae were collected to perform exposure experiments (Figure 1).

### 2.2. Exposure of PAHs-Contaminated Filaments to Larvae

The stock solutions of PAHs, BaP and Phe were prepared in 1.0 mg/mL in DMSO. The stock solutions were then stored in amber glass vials at 4 °C until further use. Prior to the experiment, the microplastic filaments were thoroughly cleaned and air-dried to ensure no contaminants were present on the surface. The microplastic filaments were then soaked in the seawater solutions of PAHs solubilized by DMSO with continuous vibration for 24 h to ensure even distribution of the PAHs on the filament surface. After the 24 h soaking period, the filaments were removed from the PAHs solutions and air-dried in a fume hood to allow the DMSO to evaporate, leaving only the PAHs on the filament surface. A total of 10 ind/mL concentration of healthy mussel larvae at the pediveliger stage was obtained by collecting them from a healthy and uncontaminated source. The larvae were then acclimated to the experimental conditions (18 °C, 26‰ salinity) for 24 h before the experiment began. A 1600 mL volume of filtered seawater was used as the experimental medium for each exposure group. The seawater was continuously aerated using a hose connected to an airstone, ensuring an aeration rate of 10 L/min for a volume of 1600 mL of seawater. The temperature of the water was controlled within a climatic chamber, maintaining a constant temperature of 18 °C. The salinity and pH levels of the seawater were measured and adjusted to 26‰ and 8.0, respectively, during the preparation phase. These parameters were consistently monitored throughout the entire duration of the experiment to ensure stable conditions for optimal mussel larval growth and survival. The PAHs-contaminated filaments were then introduced into the exposure chambers containing mussel larvae. Specifically, 5 10-cm-long filaments contaminated with PAHs were immersed in 1600 mL of seawater, maintaining a larval concentration of 10 ind/mL. The larvae were exposed to the contaminated filaments for five days. Each day was associated with an individual exposure container, necessitating the use of five identical setups to conduct the five-day experiment. Furthermore, all experiments were replicated in triplicate to ensure robustness of the results. Throughout the exposure period, the number of dead individuals was recorded each day. To achieve consistent and representative sampling, the exposure water was meticulously mixed to ensure homogenous dispersion of the larvae. Random sampling of the larvae was performed by utilizing a fine mesh sieve, followed by microscopic examination of the collected specimens to ascertain their viability and determine the precise count. Additionally, two experimental groups were exposed to corresponding concentrations of DMSO to exclude any potential effects of DMSO on the experimental results. The experimental conditions for these groups were the same as the other groups, but did not include PAHs. The design and procedural details of the exposure experiment are presented in Table 1. After the five-day exposure period, the larvae and filaments were carefully collected using a fine mesh sieve, taking care not to damage the surviving larvae. The PAHs concentration in both the surviving larvae and the filaments was measured, to assess the degree of PAHs transfer from the contaminated filaments to the larvae during the exposure period. 

### 2.3. Sampling and PAHs Analysis

Mussel larvae were freeze-dried, ground and weighed. The plastic filaments were freeze-dried and weighed to determine their initial mass before exposure. A 1000 mL of seawater was filtered with 0.22 μm organic system filter membrane. A 30 mL of dichloromethane liquid was added to the filtered seawater for liquid extraction, and the organic layer was collected as the extracted sample. The dry larvae, filaments and seawater samples were prepared for further processes, respectively. Before the extraction process, an internal standard mixture consisting of Phenanthrene-d_10_ and benzopyrene-d_10_ was added to the samples as the internal standard to correct for any losses that may occur during the extraction and analysis process. This internal standard would also help in quantifying the concentration of the target PAHs in the samples. The samples were then subjected to a Soxhlet extraction, a widely used technique for the extraction of semi-volatile organic compounds (SVOCs) from solid matrices. A total of 60 mL of dichloromethane, an efficient organic solvent, was added to a round-bottomed flask, and the sample was extracted by Soxhlet for 24 h. This process ensures that the PAHs are efficiently extracted from the samples and are available for further analysis. After the extraction process, the sample was concentrated in a rotary evaporator to 1 mL. The rotary evaporator was used to remove the excess solvent from the extracted sample, leaving behind a concentrated solution containing the PAHs of interest. This step is crucial as it reduces the volume of the sample, making it easier to analyze and allowing for a more precise measurement of the PAHs concentration. Following the concentration step, the extracted samples were subjected to a purification process using silica gel column chromatography. This step helps remove any interfering compounds, ensuring that the final analysis accurately measures only the target PAHs. The purified samples were then eluted with a suitable solvent mixture and collected for further analysis.

The PAHs concentrations of the samples, specifically BaP and Phe, were measured by Gas chromatography–mass spectrometry (GC–MS). Before analyzing the samples, the GC–MS instrument was calibrated using a series of PAHs standards with known concentrations. This calibration curve allowed for the accurate quantification of the PAHs in the samples based on their respective peak areas in the chromatograms. The samples were then injected into the GC–MS system, and the instrument conditions were set according to the procedure and quality control parameters described in our previous study [12].

### 2.4. Statistical Analysis

All data are expressed as mean ± SD and were analyzed by one-way analysis of variance (ANOVA) using SPSS software. Differences were considered statistically significant at *p* < 0.05.

## 3. Results and Discussion

### 3.1. The Toxic Effects of Contaminated Microplastic Filaments on Larval Mussels

**Figure 2:**
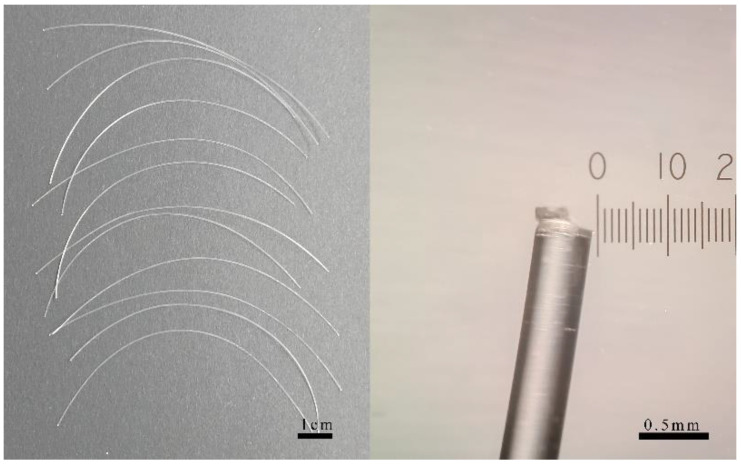
Display of PE filaments (**left**) and observation of tail end (**right**) before PAHs soak.

**Figure 3:**
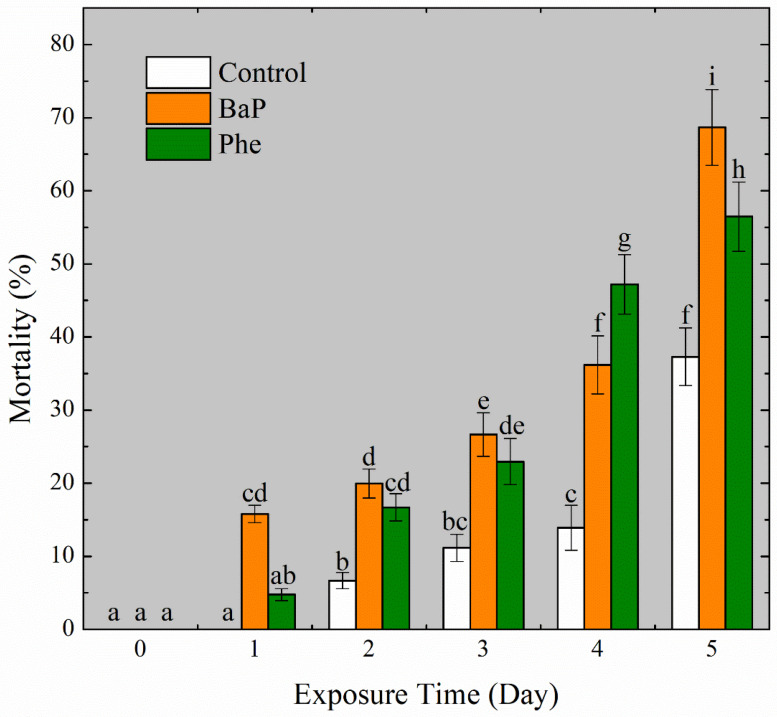
Mortalities of mussel larvae, those which were exposed to PAHs-contaminated microplastic filaments for five days. Letter labels (a, b, c, etc.) were used to indicate significant differences between the groups. The statistical analysis method was ANOVA with a significance level of 0.05.

A total of 12 microplastic filaments with 10.03 ± 0.05 cm lengths and 0.38 ± 0.005 mm diameters were soaked in the PAHs solutions (200 μg/L BaP and 500 μg/L Phe, in sea water, solubilized by DMSO) with vibration for 24 h (Figure 2). The chosen concentration was based on the results of a pre-experiment, which showed a mortality rate of around 50% within 5 days. The PAHs concentrations on the filaments surface were measured by GC–MS, but they were not detected due to a detection limit of 50 μg/L. The concentrations of both PAHs on filaments were less than 2.54 µg/g for BaP and 3.56 µg/g for Phe after calculation. The low level of PAHs adsorbed on the filaments were exposed in a 1600 mL of artificial seawater medium. Therefore, the final concentrations of both PAHs in medium were less than 130.2 ng/L of BaP and 182.5 ng/L of Phe. The pelagic larvae were exposed in the medium which contained microplastic filaments contaminated with PAHs for five days. The mortalities of larvae were recorded in each 24 h period. The dead individuals were not removed from the exposure medium during the whole period. The mortality values represented increasing trends along with exposure time (Figure 3). The mortality rates of the DMSO group (0, 0, 5.84%, 11.42%, 14.51% and 35.81% for each day, respectively) did not significantly differ from those of the control group (*p* > 0.05), indicating that DMSO did not have a significant effect on the mortality rates in this study.

In the whole period of exposure, the concentrations of BaP and Phe in larval bodies were measured by day and undetected based on the detection limit. The concentrations of PAHs in larvae were under 6.25 ng/ind for both BaP and Phe. The mortalities of the control group without PAHs exposure grew over time too. The difference of mortalities between the control group and blank group without filaments or PAHs was insignificant (*p* > 0.05). The conditions of culture have not yet been refined since environmental stress was reflected on the mortalities of the control group. Comparing to the control group, the toxic effects of PAHs were measured obviously in exposure groups. 

The exposure concentrations of two PAHs were higher than the average level of average level of PAHs in surface seawater, which is 8.5 ng/L [13]. The experimental design was to simulate the enrichment of HOCs in the marine environment. High concentrations of HOCs on microplastics have been widely reported in seawater [14,15,16]. It has been demonstrated that microplastics can act as vectors for HOCs and other contaminants, leading to their bioaccumulation in marine organisms [17]. The presence of contaminants on microplastics can also exacerbate the adverse effects of microplastics themselves [18]. However, the recent study’s ecotoxicological experiments were focused on the digestive tract route of exposure for plain and contaminated microplastics. The morphological characteristics of microplastics are commonly particles, beads and fibers on a small size-scale, which can be eaten by organisms [19,20,21]. Therefore, the “non-digestive tract route of exposure” remain little concerned. In the present study, in vitro surface contact could be the dominant pathway to transport PAHs from filaments to larvae. In the saltwater exposure medium, the desorption of PAHs from filaments was weak, and the larvae may have been exposed to PAHs when they came into contact with the contaminated filaments. Mussels in the pediveliger larval period creep onto the surface of solid objects, such as rocks, large filamentous algae, waste plastics and ship hulls, until they find a suitable position to attach by byssus threads [22]. Attachment was not observed in our study, but the creeping behavior could occur, leading to in vitro surface contact.

Some studies suggest that the attachment and ingestion of microplastics by marine organisms can increase their exposure to contaminants [23,24]. Furthermore, the exposure to PAHs-contaminated microplastics may result in a variety of toxicological effects in marine organisms, including oxidative stress, immunotoxicity and altered reproductive and developmental processes [18]. The observed increase in larval mortality over time in our study may be attributed to these toxic effects, as well as the potential for bioaccumulation and biomagnification of PAHs within the larvae [25].

Additionally, the toxic effects of PAHs on marine organisms are often influenced by various factors, including the type and concentration of PAHs, as well as the specific species and life stage of the exposed organism [26]. In our study, the exposure concentrations of BaP and Phe were higher than the average level of PAHs in surface seawater, which may have contributed to the observed toxic effects. Further research is needed to better understand the combined effects of PAHs and other contaminants in the context of microplastic exposure on larval mussels and other marine organisms.

### 3.2. Ecotoxic Risk of Contaminated Rope on Larvae in Mussel Aquaculture

In mussel aquaculture, pediveliger larvae settled on the surface of a plastic board in the breeding factory and transferred onto the surface of a rope as the final substrate. The larvae grew to juvenile mussels with strong byssuses, and the rope was hung upside down in the sea using a foam floater for years. Historically, rope was made from hemp fibers but needed to be replaced frequently due to biodegradation. However, with the wide use of the durable plastic rope, the replacement frequency has declined to a very low level. The reusable plastic rope can absorb HOCs in seawater for years and become a contamination vector [27]. The risk of contaminated ropes on mussels is still not of great concern. The potential implications of contaminated ropes in mussel aquaculture extend beyond the immediate effects on larval survival. For instance, sublethal exposure to HOCs during the larval stage may cause long-term physiological and behavioral changes in mussels, affecting their overall fitness and ability to reproduce. This could, in turn, have cascading effects on the population dynamics of mussels and the stability of the aquaculture industry.

Mussels were attached to the rope by their byssuses, and they were considered to be the dominant contaminants transferring bridge. By comparison, under contaminants exposure, larvae are more sensitive to chemicals than adult mussels [28]. Because of this, we built the experimental system to simulate the response of larvae to contaminated filaments in the present study. The results indicated that plastic filaments would transfer HOCs absorbed before to larval mussels, leading to a certain level of death. A high mortality of larvae in the earlier stages of aquaculture in sea will significantly affect the production of adult mussels. Additionally, HOCs will be transferred to the adult mussels from the contaminated plastic ropes, leading to food quality problems, which will cause a health risk to humans. In this regard, the risks posed by contaminated ropes not only impact the aquaculture industry but also have broader implications for public health and the environment. Therefore, it is crucial to develop and implement strategies that minimize the presence of HOCs in mussel aquaculture, such as more rigorous monitoring and management of ropes, as well as the adoption of alternative materials with lower HOC absorption potential.

### 3.3. Pollutant Transfer Process of Toxic Effects of Contaminated Plastic Filaments on Mussels

Microplastic filaments, which are commonly used in aquaculture operations, have been shown to absorb and accumulate organic contaminants such as PAHs from the surrounding environment. The adhesion of these contaminants to the surface of plastic filaments is a key factor in the transfer of contaminants to mussels and their larvae. Several studies have investigated the adhesion of organic contaminants to plastic surfaces. For example, Abbasi et al. [29] demonstrated that PAHs can strongly adsorb to the surface of microplastics, with the extent of adsorption varying depending on the type of plastic and the specific PAH compound. Similarly, Yu et al. [30] found that PAHs can adsorb to both virgin and aged microplastics, with the adsorption capacity increasing with increasing hydrophobicity of the plastic. The adhesion of contaminants to plastic surfaces can also be affected by environmental conditions. Additionally, temperature and salinity can impact the adsorption of HOCs to microplastics, with higher temperatures and salinities generally resulting in increased adsorption [31]. Overall, the adhesion of organic contaminants to plastic filament surfaces is a complex process that is influenced by a variety of factors. However, it is clear that microplastics can serve as a source of contaminants in the marine environment, with the potential to impact the health of aquatic organisms.

The attachment of mussels and their larvae to plastic filament surfaces is another important factor in the transfer of contaminants from microplastics to these organisms. Mussels are filter feeders that can accumulate contaminants from the water column, while their larvae are pelagic and rely on attachment to a suitable substrate for settlement and growth. The attachment of mussels and their larvae to plastic surfaces can be affected by various environmental factors. For instance, the presence of biofilms or other fouling organisms on surface can affect the attachment of mussels [32]. Additionally, the physical properties of the plastic, such as its texture and roughness, can impact the attachment of organisms [33]. The attachment of mussels and their larvae to plastic filament surfaces is an important factor in the transfer of contaminants from microplastics to these organisms. 

The transport of organic contaminants from plastic filaments to mussels is a complex process that involves several steps, including the desorption of contaminants from the plastic surface, their transport through the water column and their uptake by mussels. Several studies have investigated the transport of organic contaminants from microplastics to mussels. For example, PAHs can desorb from microplastics and become bioavailable to mussels, with the extent of desorption depending on the type of plastic and the specific PAH compound [34]. Von Moos et al. [35] found that mussels exposed to microplastics containing PAHs exhibited elevated levels of these contaminants in their tissues, indicating uptake of contaminants from the microplastics. The transport of contaminants from microplastics to mussels can be influenced by various environmental factors. The presence of other chemicals or dissolved organic matter in the water can impact the transport of contaminants [25]. Additionally, the size and shape of the microplastics can affect their transport through the water column and their uptake by mussels [36]. The mechanism of transport of contaminants from microplastics to mussels is still not fully understood. However, it is clear that microplastics can serve as a source of contaminants to mussels and other aquatic organisms, with the potential to impact their health and the health of the surrounding ecosystem.

In summary, the mechanism of toxic effects of PAHs-contaminated plastic filaments on mussels involves adhesion of organic contaminants on plastic filament surfaces, attachment of mussels and their larvae to plastic filament surfaces, and transport of organic contaminants from plastic filaments to mussels. Adhesion and attachment can facilitate the transfer of contaminants from plastic filaments to mussels, while environmental factors such as water chemistry and microplastic characteristics can influence the transport and uptake of contaminants. This research highlights the importance of understanding the mechanisms underlying the impacts of microplastics and associated contaminants on aquatic organisms, and the need for continued efforts to reduce plastic pollution in our oceans and waterways.

## 4. Conclusions

In this study, we investigated the toxic effects of PAHs-contaminated microplastic filaments on larval mussels *Mytilus coruscus* through in vitro surface contact exposure. Our results revealed a significant increase in mortality of mussel larvae over time, with 68.63% mortality for BaP and 56.45% for Phe after 5 days of exposure. Surface contact was identified as the dominant pathway for the transfer of PAHs from contaminated filaments to larvae. Our findings suggest that the risk of contaminated plastic ropes transferring HOCs to larvae in mussel aquaculture should be taken seriously. Furthermore, our study highlights the importance of understanding the mechanisms underlying the impacts of microplastics and associated contaminants on aquatic organisms. We recommend further investigation into the pathways of HOCs transferring from contaminated plastic ropes to juvenile and adult mussels by byssus threads in mussel aquaculture.

In conclusion, our research demonstrates the potential for significant adverse effects of PAHs-contaminated microplastic filaments on larval mussels, which may have implications for the aquaculture industry, public health and the environment. Therefore, targeted strategies for mitigating the impacts of microplastic pollution in marine environments, including rigorous monitoring and management of ropes, as well as the adoption of alternative materials with lower HOC absorption potential, are urgently needed. Further studies examining the toxic effects of contaminated microplastics on marine organisms across various life stages and species, as well as the potential for long-term impacts on ecosystem health and function, are also warranted.

## Figures and Tables

**Table 1:** Procedural details of the exposure experiment.

Steps	Groups			
	Control	DMSO	BaP	Phe
1: Contaminants solution	one liter seawater	one-liter DMSO solution in seawater (500 μL/L)	BaP stock solution prepared in DMSO (1.0 mg/mL); one-liter BaP seawater solution prepared (200 μg/L, 200 μL stock solution used)	Phe stock solution prepared in DMSO (1.0 mg/mL); one-liter Phe seawater solution prepared (500 μg/L, 500 μL stock solution used)
2: Contaminants adsorption	5 10-cm-long filaments soaked in above solutions for 24 h, removed from solutions and air-dried			
3: Exposure	the 5 dried filaments exposed in larvae suspension (10 ind/mL) in 1600 mL seawater for 5 days			

## Data Availability

The data presented in this study are available upon request from the corresponding author.
